# Synthesis and Biological Activity of Isoflavone Derivatives from Chickpea as Potent Anti-Diabetic Agents

**DOI:** 10.3390/molecules200917016

**Published:** 2015-09-17

**Authors:** Pengshou Li, Xiaojuan Shi, Ying Wei, Lingling Qin, Wen Sun, Guangyuan Xu, Tunhai Xu, Tonghua Liu

**Affiliations:** 1 School of Chinese Pharmacy, Beijing University of Chinese Medicine, Beijing 100029, China; E-Mails: 15501051852@163.com (P.L.); 18810959032@163.com (X.S.); weimozhuo@163.com (Y.W.); 2 Health Cultivation Laboratory of the Ministry of Education, Beijing University of Chinese Medicine, Beijing 100029, China; E-Mails: qllqxtslx@126.com (L.Q.); sunweng00897@163.com (W.S.); xuguangyuan102@126.com (G.X.); Thliu@163.com (T.L.)

**Keywords:** chickpea, isoflavones, derivative synthesis, anti-diabetic activity

## Abstract

A set of novel isoflavone derivatives from chickpea were synthesized. The structures of derivatives were identified by proton nuclear magnetic resonance (^1^H-NMR), carbon-13 (^13^C)-NMR and mass spectrometry (MS) spectral analyses. Their anti-diabetic activities were evaluated using an insulin-resistant (IR) HepG2 cell model. Additionally, the structure-activity relationships of these derivatives were briefly discussed. Compounds **1c**, **2h**, **3b**, and **5** and genistein exhibited significant glucose consumption-enhancing effects in IR-HepG2 cells. In addition, the combinations of genistein, **2h**, and **3b** (combination 6) and of **3b**, genistein, and **1c** (combination 10) exhibited better anti-diabetic activity than the individual compounds. At the same dosage, there was no difference in effect between the combination 10 and the positive control (*p* > 0.05). Aditionally, we found the differences between the combination 10 and combination 6 for the protective effect of HUVEC (human umbilical vein endothelial cells) under high glucose concentration. The protective effects of combination 10 was stronger than combination 6, which suggested that combination 10 may have a better hypoglycemic activity in future studies. This study provides useful clues for the further design and discovery of anti-diabetic agents.

## 1. Introduction

With further research into the mechanisms of various diseases, it is understood that no “Magic Bullets” exist for curing most diseases. Many diseases with high incidence rates such as diabetes, heart disease, arthritis, asthma, depression and cancer are related to many changing pathological factors [1,2,3]. However, current research and drug development have generally been limited to one-target, one-model drugs. In the other words, the goal is to identify highly selective drugs that act on a single target [4]. However, although research over the past 20 years had focused on identifying highly selective target ligands, the proportion of successful drug candidates tested in clinical trials had not increased but rather had decreased. This phenomenon prompted us to question the mode of drug development [5]. With the development of molecular biology, genomics, and systems biology, the concept of multicomponent drugs has been proposed. Multicomponent drug regimens differ from single target drugs from compounds containing only one compound; these regimens contain a variety of compounds that exert synergistic pharmacological effects, and such drugs can simultaneously interact with multiple targets. The total effect is often greater than the effect of a single compound; thus, a better therapeutic effect can be achieved.

Chickpea (*Cicer arietinum* L.) is widely used in Uygur and exerts favorable hypoglycemic effects both as a dietary or medicinal supplement. According to recent studies, isoflavones extracted from chickpea exhibit favorable hypoglycemic activity [6]; however, these reports are limited to studies of active components. In addition, previous research on the hypoglycemic activity of the compounds and hypoglycemic activity optimization based on structural modifications is not sufficient. Regarding isoflavonoids extracted and isolated from chickpea and based on the multiple target effect model of traditional Chinese medicine, studies on the hypoglycemic activity of combinations of parent compounds and their derivatives are lacking. Such studies are required to develop and utilize active compounds in chickpea.

**Figure 1:**
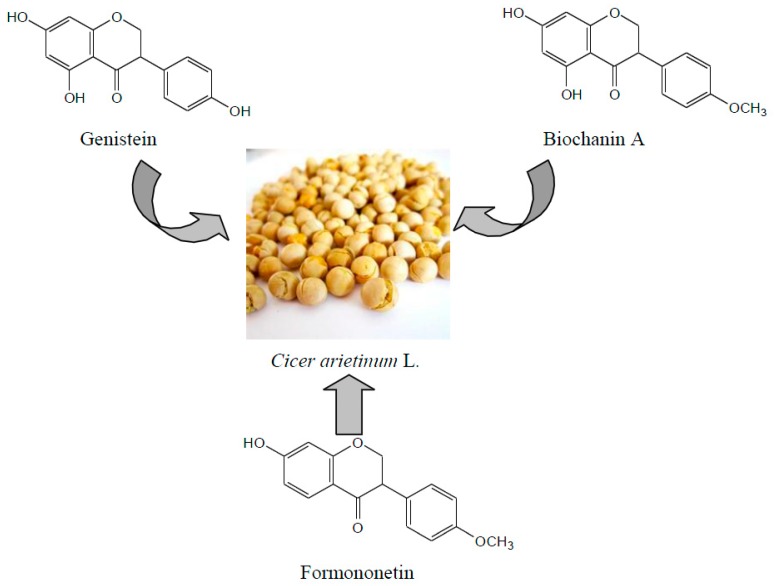
The structures of genistein, biochanin A, and formononetin.

Genistein from chickpea significantly decreased serum insulin levels, total cholesterol, triglycerides, low-density lipoprotein cholesterol, high-density lipoprotein cholesterol, and the ratio of high-density lipoprotein cholesterol to low-density lipoprotein cholesterol, improved glucose tolerance, and reduced serum liver glycogen and muscle glycogen in type 2 diabetic rats [7]. Genistein can be used not only to treat type 2 diabetes but also to stimulate insulin release in type 1 diabetes [8]. Because of isoflavones structure and the effect of surrounding phenolic hydroxyl, genistein has low bioavailability. The combination of formononetin and hanfangichin B exerted favorable hypoglycemic effects in rats with streptozotocin-induced diabetes, and formononetin reduced blood glucose by stimulating insulin release [9]. In addition, biochanin A is also a major isoflavone component in chickpea. Although there are few reports of hypoglycemic activity, hypoglycemic activity can be obtained via structural modification, using the same isoflavone skeleton structure with genistein and biochanin A, thus expanding the hypoglycemic applications of chickpea isoflavonoids. Therefore, based on structural modifications of genistein, biochanin A, and formononetin derived from chickpea and combinations of parent compounds and their derivatives, we studied the hypoglycemic activity of both individual compounds and combinations of compounds. These results provide a reference for the development of drugs that exert hypoglycemic activity. The structures of genistein, biochanin A, and formononetin are shown in Figure 1.

## 2. Results and Discussion

### 2.1. Synthesis

In the current study, a set of novel derivatives were synthesized, among which compounds **1d**–**1e**, **2b**–**2j**, **3c**–**3d**, **4b** and **5** have not been previously reported.

#### 2.1.1. Sulfonate Derivative Formation

**Scheme 1:**
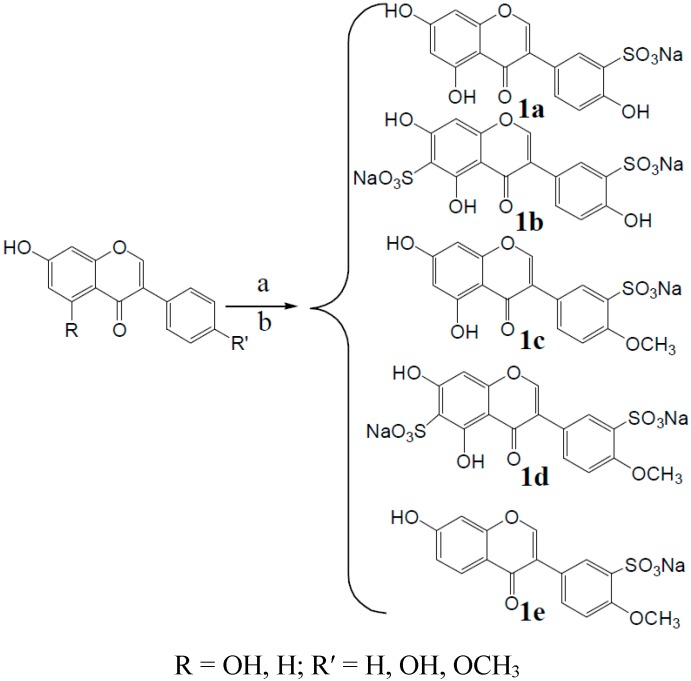
Synthesis of compounds **1a**–**1e**.

Because of their isoflavone skeletons and the influence of hydroxyl groups, the lipid and water solubility of these isoflavones is poor. This characteristic lead to low bioavailability and limit their widespread applications. Some researchers attempted to improve the water solubility of these isoflavones using physical methods, but the effect was not obvious. Structural modification of the sulfonic acid sodium of genistein, biochanin A, and formononetin were used to improve their water solubility. The synthetic routes to derivatives **1a**–**1e** are shown in Scheme 1.

*Reagents and conditions*: (**a**) concentrated sulfuric acid; (**b**) NaCl, room temperature (r.t.).

#### 2.1.2. Structural Modification: Isopropylation

**Scheme 2:**
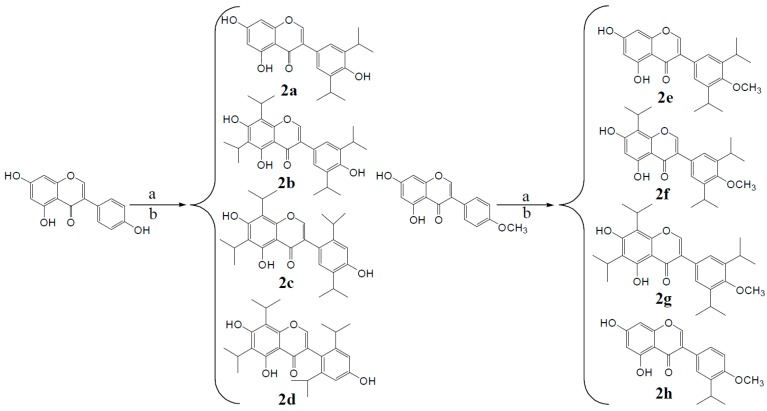

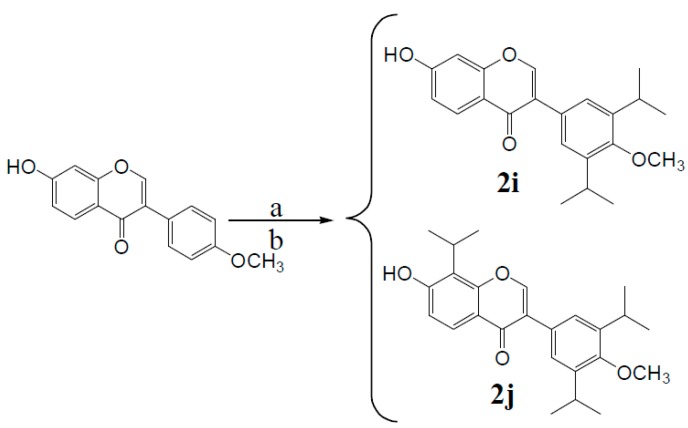
Synthesis of compounds **2a**–**2j**.

Adding another lipid soluble group to the parent isoflavones can increase lipid solubility of the compounds and increase the ability of the compound to pass through biomembranes and to reach the target tissues and organs. The synthetic routes to derivatives **2a**–**2j** are shown in Scheme 2.

*Reagents and conditions*: (**a**) (CH_3_)_2_CHOH, H_2_SO_4_; (**b**) 60 °C, 3 h.

#### 2.1.3. Split with Other Small Molecules

**Scheme 3:**
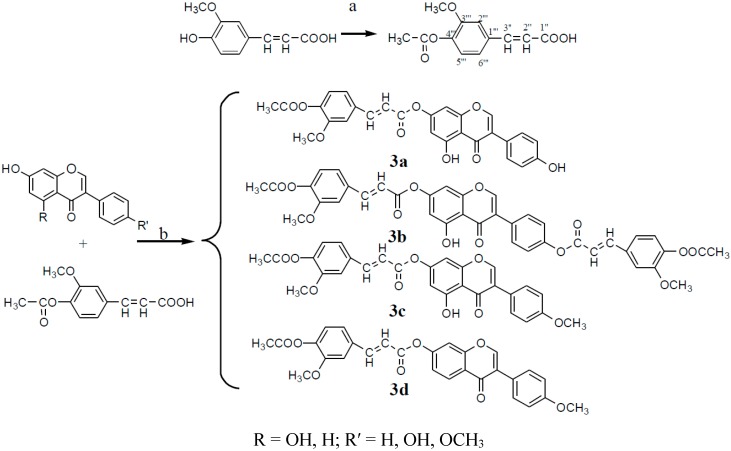
Synthesis of compounds **3a**–**3d**.

The parent isoflavones were esterified with acetyl ferulic acid, which has strong effects regarding many aspects of diabetes. This structural modification may improve their selectivity and efficacy. The synthetic routes to derivatives **3a**–**3d** are shown in Scheme 3.

*Reagents and conditions*: (**a**) (CH_3_CO)_2_O, Py, reflux 4 h; (**b**) DCC, Py, reflux, 80 °C, 4 h.

#### 2.1.4. The Introduction of Fluorine (F) Elements

**Scheme 4:**
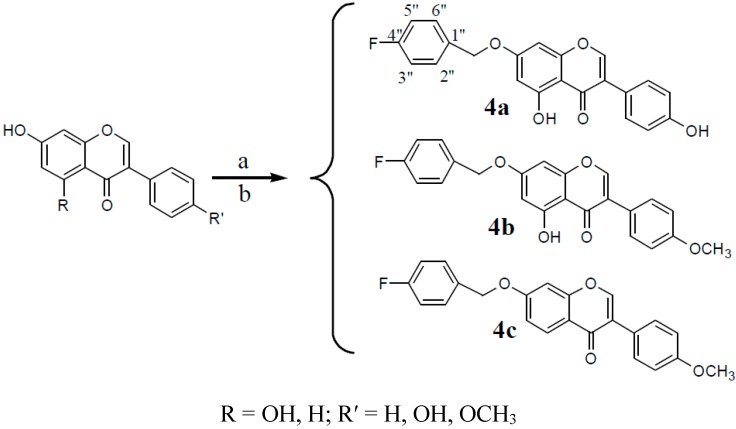
Synthesis of compounds **4a**–**4c**.

Recently, organofluorine compounds have played an important role in the study and development of drugs for the treatment of diabetes. For example, fluorine-containing drugs such as Januvia and the aldose reductase inhibitor Sorbinil have been used to treat type II diabetes. Therefore, compounds containing fluorine, which were expected to improve hypoglycemic activity, were synthesized in this study. The synthetic routes to derivatives **4a**–**4c** are shown in Scheme 4.

*Reagents and conditions*: (**a**) 4-fluorobenzyl bromide, CH_3_ONa; (**b**) CH_3_OH, CH_2_Cl_2_, reflux 20 h.

#### 2.1.5. Coordination with Trace Elements

**Scheme 5:**
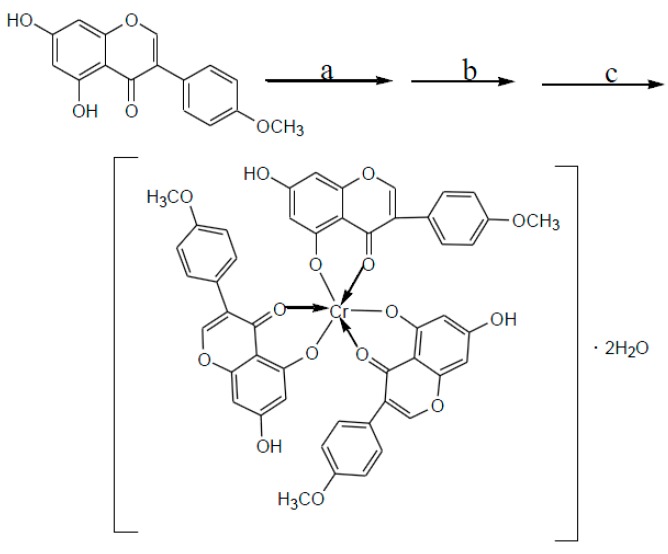
Synthesis of compound **5**.

Chromium (Cr^3+^) is an essential trace element in the human body and is required for glucose and lipid metabolism. Cr^3+^ impacts the structure and function of insulin and the status of target tissues and cells significantly. A lack of chromium in the body leads to the dysregulation of glucose and lipid metabolism and promotes atherosclerosis, diabetes, hyperlipidemia and other symptoms. Previous studies had shown that diabetic patients were often chromium deficient. Isoflavones may form complexes with chromium, which could reduce toxicity of inorganic chromium and increase the degree of absorption [10] in diabetic patients. This may lead to better hypoglycemic effects. The synthetic route to derivative **5** is shown in Scheme 5.

*Reagents and conditions*: (**a**) CH_3_CH_2_OH, 60 °C, 1 h; (**b**) CH_3_COOCr, 80 °C,18 h; (**c**) maturing 48 h.

### 2.2. Biological Activity

#### 2.2.1. Study of the Hypoglycemic Activity of Individual Compounds

Many anti-diabetic isoflavonoids have been reported [11,12,13]. However, these isoflavonoids have exhibited weak activity, as revealed by their high doses, compared with drugs currently on the market. The isoflavonoids in chickpea are known for their anti-hyperglycemic effects, and their hypoglycemic activity had been previously reported [1,14]. For the first time, we synthesized a series of isoflavonoid (genistein, biochanin A, and formononetin) derivatives and studied the hypoglycemic activities of these derivatives. We identified that these compounds induced significant anti-diabetic activities compared with the commercially available drug metformin.

**Figure 2:**
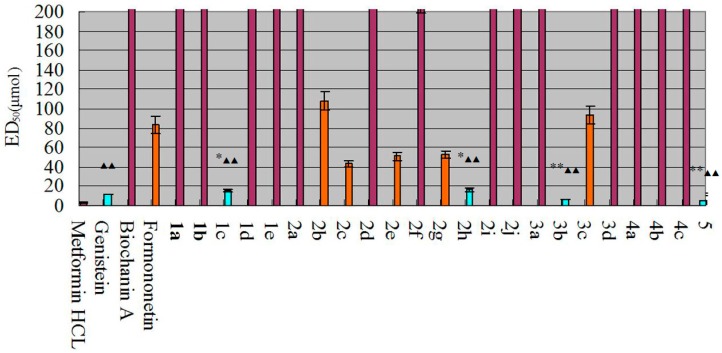
Hypoglycemic activity of the compounds. The differences between these compounds and genistein were statistically significant (denoted by “*” or “**” for *p* < 0.05 or *p* < 0.01, respectively). The differences between these compounds and metformin hydrochloride were statistically significant (denoted by “^▲^” or “^▲▲^” for *p* < 0.05 or *p* < 0.01, respectively).

In this study, an insulin-resistant (IR) HepG2 cell model [15,16,17] was used to evaluate the anti-hyperglycemic effects of the isoflavonoids derivatives. The individual compounds **1c**, **2h**, **3b**, **5** and genistein all exerted significant glucose consumption-enhancing effects in the IR-HepG2 cells. The hypoglycemic activity of each individual compound is shown in Figure 2.

Compounds with an ED_50_ < 20.0 μmol were considered to exert strong hypoglycemic activity; such compounds are shown in bright blue. The ED_50_ value of genistein was approximately 20 μmol; thus, genistein was included into this group. The effects of compounds **1c**, **2h**, **3b**, and **5** were superior to those of genistein. Compounds corresponding to 100 μmol > ED_50_ > 20.0 μmol were considered to have weaker hypoglycemic activity; these compounds are shown in orange. Such compounds could be used as substrates for further structural modification. The ED_50_ value of compound **2b** was approximately 100 μmol; thus, compound **2b** was included in this group. Compounds with an ED_50_ > 100.0 μmol were considered to exert very weak hypoglycemic activity and were not considered for further evaluation; these compounds are shown in purple.

##### Structure-Activity Relationship Analysis

The hypoglycemic activity of genistein was significantly better than that of biochanin A (*p* < 0.01), indicating that 4′-OH substituted by methoxyl greatly decreased the hypoglycemic activity and that 4′-OH was a strong active group. The activity of formononetin and biochanin A showed that activity was greatly reduced if 5-OH existed, indicating that 5-OH was a group that prevent producing strong active.

Due to the derivatization of sodium sulfonate, compound **1c** exhibited strong activity. Compared with the activity of biochanin A, the difference was significant (*p* < 0.01). The difference was also significant (*p* < 0.01) compared with other isoflavone derivatives of sodium sulfonate. This result indicates that the sodium sulfonate group in genistein decrease the hypoglycemic activity of the compounds, possibly due to the sodium sulfonate group leading to weak action of phenol hydroxyl on targets in the body. The 3′-sodium sulfonate group in biochanin A may interact with ortho-methoxy and generate more active groups.

The activities of the isopropylation-products showed that compound **2h** exhibited optimal activity, and compounds **2b**, **2c**, **2e** and **2g** exerted relatively weak activities. The other derivatives of isopropylation exerted no significant activity. Through the introduction of 5′-isopropyl, the hypoglycemic activity of biochanin A was increased. However, the activity was weakened due to the introduction of 3′-isopropyl after introduction of 5′-isopropyl. The activity was also attenuated due to the introduction of 3′,6 and 8-isopropyl after introduction of 5′-isopropyl, indicating that the introduction of too many isopropyl hinder the effect of the compounds on the target. However, compound **2f** exhibited no activity, revealing that the 6, 8-isopropyl or 6, 8-hydrogen of biochanin A is necessary when 3′,5′-isopropyl exist if we want the derivatives exhibit activity. For genistein, the activity of compound **2c** (2′,5′,6,8-isopropyl) was better than compound **2b** (3′,5′,6,8-isopropyl); moreover, compound **2d** (2′,6′,6,8-isopropyl) and **2a** (3′,5′-isopropyl)did not result in activity. So the hypoglycemic activity is: 2′,5′ > 3′,5′ > 2′,6′-isopropyl after 6,8-isopropyl were introduction. For formononetin, the hypoglycemic activity was lost due to substitution of the isopropyl. For genistein and biochanin A, when the 3′,5′,6,8-isopropyl were introduced, the activity was better when the 4′-hydrogen was replaced by a methoxyl group rather than a hydroxyl group. When the 3′,5′-isopropyl were introduced, the activity was better when the 4′-hydrogen was replaced by a methoxyl group rather than a hydroxyl group.

For genistein, substitution by acetyl ferulic acid showed that the activity was increased when the 4′,7-hydroxyl were replaced by acetyl ferulic acid compared with only 7-hydroxyl was substituted. This indicated that more acetyl ferulic acid increased the target binding efficiency. The hypoglycemic activity of compound **3b** was better than genistein and the difference was significant (*p* < 0.01). For biochanin A, the hypoglycemic activity of compound **3c** was better than biochanin A and the difference was also significant (*p* < 0.01). In addition, for compounds **3a** and **3c**, when the 7-hydroxyl was substituted, the activity was better when the 4′-hydrogen was replaced by a methoxyl group rather than by a hydroxyl group. Thus, under these structural conditions, the target binding effect is stronger when the 4′-hydrogen is replaced by a methoxyl group rather than by a hydroxyl group. In addition, for formononetin, substitution of the 7-OH with acetyl ferulic acid induced a loss in activity.

For the derivatives that were replaced by 4-fluorobenzyl bromide, 7-hydroxyl was substituted for three starting material. However, all the derivatives losted hypoglycemic activity, indicating that the 4-fluorobenzyl bromide was not required to increase the activity of hypoglycemic activity.

The hypoglycemic activity of compound **5** was stronger, indicating that the introduction of chromium greatly enhanced the hypoglycemic activity of the compound.

The structure-activity relationship analysis are shown in Table 1.

**Table 1:** Structure-activity relationship analysis.

The Hypoglycemic Activity	Structure-Activity Relationship	
	Active Group	Non Active Group
Genistein > Formononetin > Biochanin A	4′-OH is a strong active group	4′-OCH_3_ and 5-OH were groups that prevent producing strong active
Genistein > **1c** > Formononetin > **1a**, **1b**, **1d**, **1e**, Biochanin A	3′-sodium sulfonate group in biochanin A	3′ or 3′,6-sodium sulfonate groups in genistein; 3′-sodium sulfonate group in formononetin
Genistein > **2h** > **2c** > **2e**> **2g** > Formononetin > **2b** > **2a**, **2d**, **2f**, **2i**, **2j**, Biochanin A	BiochaninA: only 5′-isopropyl; 6,8-isopropyl or 6,8-hydrogen of biochanin A is necessary when 3′,5′-isopropyl exist.	Biochanin A: 3′,5′,8- isopropyl Genistein: all the isopropyl replacement. Formononetin: same to Genistein.
**3b** > Genistein > Formononetin > **3c** > **3a**, **3d**, Biochanin A	4′,7-acetyl ferulic acid in genistein; 7-acetyl ferulic acid in biochanin A	7-acetyl ferulic acid in genistein; 7-acetyl ferulic acid in formononetin
Genistein > Formononetin > Biochanin A > **4a**, **4b**, **4c**		7-hydroxyl was substituted by 4-fluorobenzyl bromide in three starting material
**5** > Genistein > Formononetin > Biochanin A	Cr^3+^	

#### 2.2.2. The Hypoglycemic Activity of Combined Compounds

**Figure 3:**
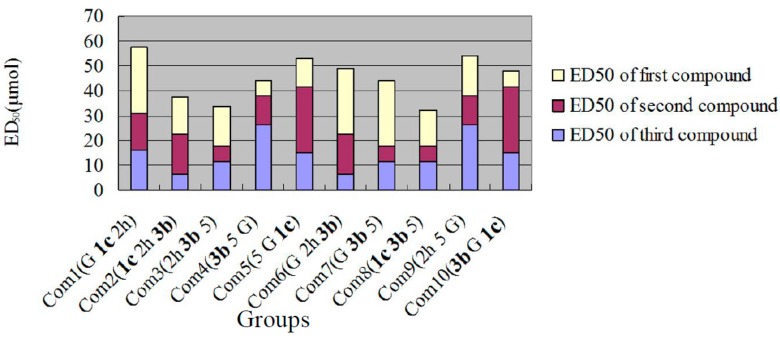
Combinations and activity superposition.

We selected the derivatives with the best hypoglycemic effects and genistein (G) for use in combinations. Each combination is shown in Figure 3. The y-axis indicates the sum of the ED_50_ values of the compounds.

**Figure 4:**
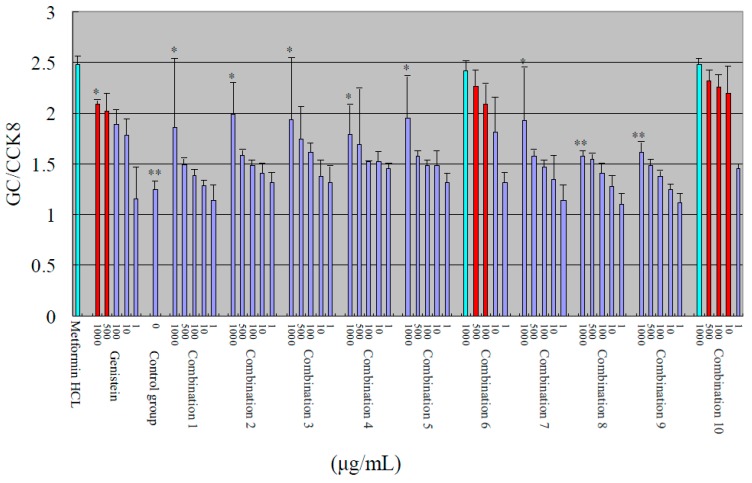
Hypoglycemic activities of combinations. The differences between combination 6, combination 10 and other combinations were statistically significant (*p* < 0.05 or *p* < 0.01, denoted by “*” or “**”, respectively).

The hypoglycemic activities of the combinations evaluated according to glucose consumption (GC)/CCK8 are shown in Figure 4.

Figure 4 shows that the hypoglycemic effects of the maximum concentration of combination 6 and combination 10 (shown in bright blue) were similar to the effect of metformin hydrochloride in the positive control group; the difference was not significant (*p* > 0.5). The effect of the maximum concentration of combination 6 and combination 10 was better than that of the other combinations. At the same dosage (500 µg/mL) as the positive control (3.02 µmol), no difference was found in the effects between combination 10 (**3b** = 0.24 μmol, G = 0.43 μmol, **1c** = 0.62 μmol, total 1.29 μmol) and the positive control (*p* > 0.05).

The true activities differed from the activities calculated by simple addition. Combination 3 and combination 8 exhibited the best hypoglycemic effects based on simple addition, whereas the actual best effect was obtained using combination 6 and combination 10. This result indicates interference by the compounds; thus, their effects on multiple target points should be considered. Genistein and the other dosages of combination 6 and combination 10 (shown in red) can be used to obtain new combinations in future studies.

#### 2.2.3. The Effect of Combined Compounds on Cell Proliferation and Toxicity

**Figure 5:**
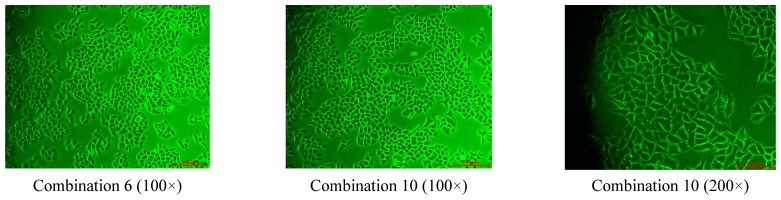

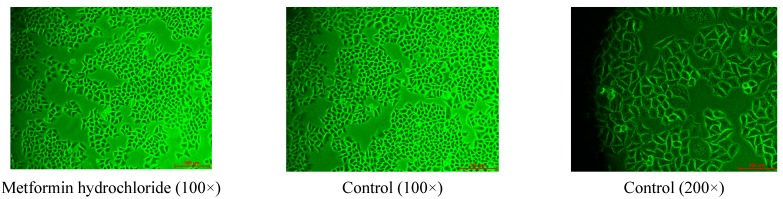
Cellular morphology.

Compared with the control group, changes in cell morphology or cell number were not found, indicating that the toxicity of the combinations was low, as shown in Figure 5.

#### 2.2.4. The Impacts of HUVEC Secrete LDH, MDA, SOD and NO under High Concentration of Glucose

**Figure 6:**
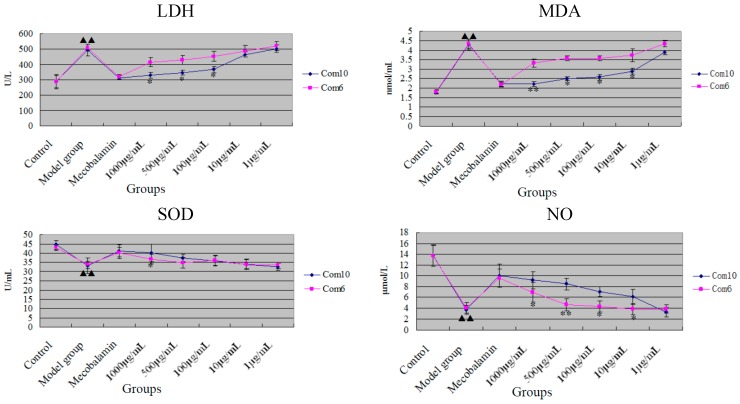
Combination 6 and combination 10 acting on human umbilical vein endothelial cells (HUVEC) under high concentration of glucose. The differences of LDH and MDA between model group and control group were statistically significant (*p* < 0.01, denoted by “^▲▲^”).The differences of NO content and SOD activity between model group and control group were statistically significant (*p* < 0.01, denoted by “^▲▲^”). The differences of LDH, MDA, NO and SOD between combination 6 and combination 10 were statistically significant (*p* < 0.05 or *p* < 0.01, denoted by “*”or “**”, respectively).

We found significant differences between combination 6 and combination 10 acting on HUVEC under high concentration of glucose. Wherein the combination 10 had better protective effects on HUVEC, and combination 6 had relatively weak protective effects. This prompted us that combination 10 would have better hypoglycemic effect through protecting HUVEC in further study. The protective effects were shown in Figure 6.

Research indicated that model group of HUVEC cultured in medium of high concentration of glucose had higher content of lipid peroxidation and produced more LDH and MDA than control group. And NO content and SOD activity was decreased compared with control group. Combination 10 could significantly reduce the amount of MDA and LDH leakage of HUVEC injured by high glucose, and it also increased the NO content and SOD activity. Compared with combination 6, the difference was obvious, which indicated that combination 10 could play a better role of protecting HUVEC.

## 3. Experimental Section

### 3.1. General Information

All reactions were monitored by thin-layer chromatography (TLC) on silica gel GF-254 plates purchased from Qingdao Haiyang Corporation. Proton nuclear magnetic resonance (^1^H-NMR) and carbon-13 (^13^C)-NMR spectra were acquired using a Bruker AV500 MHz spectrometer, and dimethylsulfoxide (DMSO-*d*_6_) was used as the solvent. Chemical shifts are reported in parts per million shift (δ value) based on Me_4_Si (δ 0 ppm for ^1^H). High-resolution mass spectra (HR-MS) were acquired using a Thermo Scientific LTQ Orbitrap XL (New York, NY, USA). Infrared (IR) analyses were conducted using a Thermo Nicolet Nexus 670 spectrometer (New York, NY, USA). Ultraviolet (UV) analyses were conducted using a UV-2450 Ultraviolet visible spectrophotometer. Melting points (m.p., uncorrected) were measured using an X-5 micro melting point apparatus (Beijing, China). Silica-gel column chromatography was performed using 300–400 mesh silica gel purchased from Qingdao Haiyang Corporation. The yields were calculated based on the last step of the reaction. All chemicals and solvents used were analytical or high-performance liquid chromatography grade.

### 3.2. Chemistry

#### 3.2.1. Synthesis of Compounds **1a**–**1e** (Considering One of the Parental Compounds as an Example)

A mixture of genistein (10.00 g, 0.037 mol) and 25 mL concentrated sulfuric acid was heated to 60 °C under stirring for 0.5 h. The mixture was then poured into 500 mL ice-cold saturated brine. A precipitate formed slowly; the precipitate formed completely after 4 h incubation. The precipitate was filtered and washed with saturated brine until the pH became neutral. The crude material was purified in a 300–400 mesh silica column (water-saturated butanol).

*3′-Sodium sulfonate genistein* (**1a**). The product was obtained as a white solid (35.2% yield); m.p. 304~305 °C. ^1^H-NMR (DMSO-*d*_6_) δ (ppm): 12.91 (s, 1H, 5-OH), 10.82 (s, 1H, 7-OH), 10.56 (s, 1H, 4′-OH), 8.36 (s, 1H, H-2), 7.71 (s, 1H, H-2′), 7.37 (d, *J* = 8.4 Hz, 1H, H-6′), 6.86 (d, *J* = 8.4 Hz, 1H, H-5′), 6.41 (s, 1H, H-8), 6.24 (s, 1H, H-6). ^13^C-NMR (DMSO-*d*_6_) δ (ppm): 180.50 (s, C-4), 164.82 (s, C-7), 162.42 (s, C-4′), 158.06 (s, C-5), 154.77 (s, C-8a), 153.81 (s, C-2), 132.01 (s, C-2′), 131.08 (s, C-3′), 128.20 (s, C-6′), 122.22 (s, C-1′), 121.55 (s, C-3), 116.84 (s, C-5′), 104.88 (s, C-4a), 99.50 (s, C-6), 94.21 (s, C-8). HRMS (ESI) *m*/*z*: 269.01752 [M − SO_3_Na − H]^−^, calcd. for C_15_H_9_O_5_ 269.04500.

*3′,6-Disodium sulfonate genistein* (**1b**). The product was obtained as a white solid (31.2% yield); m.p. 312~313 °C. ^1^H-NMR (DMSO-*d*_6_) δ (ppm): 13.27 (s, 1H, 5-OH), 12.59 (s, 1H, 7-OH), 10.63 (s, 1H, 4′-OH), 8.51 (s, 1H, H-2), 7.74 (s, 1H, H-2′), 7.38 (d, *J* = 8.4 Hz, 1H, H-6′), 6.85 (d, *J* = 8.0 Hz, 1H, H-5′), 6.21 (s, 1H, H-8). ^13^C-NMR (DMSO-*d*_6_) δ (ppm): 180.65 (s, C-4), 162.37 (s, C-7), 161.29 (s, C-4′), 155.12 (s, C-5), 154.97 (s, C-8a), 153.94 (s, C-2), 132.25 (s, C-2′), 131.04 (s, C-6′), 128.53 (s, C-3′), 122.66 (s, C-1′), 121.16(s, C-3), 116.87 (s, C-5′), 105.10 (s, C-4a), 101.86 (s, C-6), 99.52 (s, C-8). HRMS (ESI) *m*/*z*: 370.98638 [M − SO_3_Na − H]^−^, calcd. for C_15_H_8_NaO_8_S 370.98376.

*3′-Sodium sulfonate biochanin A* (**1c**). The product was obtained as a buff solid (34.3% yield); m.p. 221~222 °C. ^1^H-NMR (DMSO-*d*_6_) δ (ppm): 12.93 (s, 1H, 5-OH), 10.94 (s, 1H, 7-OH), 8.35 (s, 1H, H-2), 7.89 (s, 1H, H-2′), 7.48 (d, *J* = 8.4 Hz, 1H, H-6′), 7.02 (d, *J* = 7.9 Hz, 1H, H-5′), 6.42 (s, 1H, H-8), 6.25 (s, 1H, H-6), 3.79 (s, 3H, -OCH_3_). ^13^C-NMR (DMSO-*d*_6_) δ (ppm): δ 180.56 (s, C-4), 164.83 (s, C-7), 162.44 (s, C-4′), 158.10 (s, C-5), 156.74 (s, C-8a), 154.76 (s, C-2), 135.92 (s), 131.34 (s), 129.66 (s), 122.58 (s), 121.93 (s), 112.21 (s), 104.90 (s, C-4a), 99.51 (s), 94.23 (s), 56.06 (s, -OCH_3_). HRMS (ESI) *m*/*z*: 283.06832 [M − SO_3_Na − H]^−^, calcd. for C_16_H_11_O_5_ 283.06065.

*3′,6-Disodium sulfonate biochanin A* (**1d**). The product was obtained as a buff solid (32.3% yield); m.p. 228~229 °C. ^1^H-NMR (DMSO-*d*_6_) δ (ppm): 13.28 (s, 1H, 5-OH), 12.59 (s, 1H, 7-OH), 8.50 (s, 1H, H-2), 7.94 (s, 1H, H-2′), 7.47 (d, *J* = 8.3 Hz, 1H, H-6′), 7.06 (d, *J* = 8.5 Hz, 1H, H-5′), 6.21 (s, 1H, H-8), 3.82 (s, 3H, -OCH_3_). ^13^C-NMR (DMSO-*d*_6_) δ (ppm): 180.66 (s, C-4), 162.38 (s, C-7), 161.31 (s, C-4′), 156.73 (s, C-5), 155.15 (s, C-8a), 154.87 (s, C-2), 135.68 (s), 131.39 (s), 129.89 (s), 122.91 (s), 121.73 (s), 112.29 (s), 110.62 (s), 105.09 (s, C-4a), 99.52 (s), 56.19 (s, -OCH_3_). HRMS (ESI) *m*/*z*: 385.01812 [M − SO_3_Na − H]^−^, calcd. for C_16_H_10_NaO_8_S 384.99941.

*3′-Sodium sulfonate formononetin* (**1e**). The product was obtained as a white solid (31.7% yield); m.p. 263~264 °C. ^1^H-NMR (DMSO-*d*_6_) δ (ppm):10.88 (s, 1H, 7-OH), 8.31 (s, 1H, H-2), 7.98 (d, *J* = 8.7 Hz, 1H, H-5), 7.89 (d, *J* = 1.4 Hz, 1H, H-2′), 7.50 (dd, *J* = 8.4, 1.4 Hz, 1H, H-6′), 7.05 (d, *J* = 8.5 Hz, 1H, H-5′), 6.96 (dd, *J* = 8.8, 1.0 Hz, 1H, H-6), 6.91 (s, 1H, H-8), 3.81 (s, 3H, -OCH_3_). ^13^C-NMR (DMSO-*d*_6_) δ (ppm): 175.03 (s, C-4), 163.10 (s, C-7), 157.95 (s, C-4′), 156.40 (s, C-8a), 153.59 (s, C-2), 135.58 (s), 131.40 (s), 129.53 (s), 127.72 (s), 123.77 (s), 123.37 (s), 117.03 (s), 115.69 (s), 112.12 (s), 102.64 (s), 56.19 (s, -OCH_3_). HRMS (ESI) *m*/*z*: 267.06378 [M − SO_3_Na − H]^−^, calcd. for C_16_H_1__1_O_4_ 267.06574.

#### 3.2.2. Synthesis of Compounds **2a**–**2j** (Considering One of the Parental Compounds as an Example)

Compounds **2a**–**2j** were prepared according to the method described by Xie *et al.* [18]. Formononetin (1.0 g, 3.73 mmol) was dissolved in 16 mL 80% sulfuric acid. After the formononetin was dissolved, isopropyl alcohol (3 mL) was added to the mixture. The mixture was heated to 60 °C under stirring for 3 h, and the mixture was then poured into water (200 mL). White floc formed immediately upon the addition of water, and the precipitate formed completely after 4 h incubation. The precipitate was filtered and washed with water until the pH became neutral. The crude material was separated in a 300–400 mesh silica column (petroleum ether:ethyl acetate, 3:1).

*3′,5′-Diisopropyl genistein* (**2a**). The product was obtained as a white solid (22.1% yield); m.p. 284~285 °C. ^1^H-NMR (DMSO-*d*_6_) δ (ppm): 13.01 (s, 1H, 5-OH), 10.89 (s, 1H, 7-OH), 9.31 (s, 1H, 4′-OH), 8.26 (s, 1H, H-2), 7.17 (s, 2H, H-2′,6′), 6.38 (s, 1H, H-8), 6.23 (s, 1H, H-6), 3.33 (dt, *J* = 13.2, 6.7 Hz, 2H, -CH(CH_3_)_2_), 1.18 (d, *J* = 6.7 Hz, 12H, -CH(CH_3_)_2_). ^13^C-NMR (DMSO-*d*_6_) δ (ppm): 180.82 (s, C-4), 164.70 (s, C-7), 162.49 (s, C-4′), 158.05 (s, C-5), 154.53 (s, C-8a), 151.30 (s, C-2), 135.41 (s, C-2′,6′), 124.44 (s, C-3′,5′), 123.45 (s, C-1′), 122.51 (s, C-3), 104.97 (s, C-4a), 99.39 (s, C-6), 94.08 (s, C-8), 26.68 (s, 2C), 23.43 (s, 4C). HRMS (ESI) *m*/*z*: 353.09991 [M − H]^−^, calcd. for C_21_H_2__1_O_5_ 353.13890.

*3′,5′,6,8-Tetraisopropyl genistein* (**2b**). The product was obtained as a white solid (21.1% yield); m.p. 278~279 °C. ^1^H-NMR (DMSO-*d*_6_) δ (ppm): ^1^H-NMR (500 MHz, DMSO) δ 13.61 (s, 1H, 5-OH), 10.99 (s, 1H, 7-OH), 9.46 (s, 1H, 4′-OH), 8.24 (s, 1H, H-2), 7.17 (s, 2H, H-2′,6′), 3.58 (m, *J* = 14.0, 6.9 Hz, 1H, -CH(CH_3_)_2_), 3.50 (dt, *J* = 13.9, 7.1 Hz, 1H, -CH(CH_3_)_2_), 3.34–3.26 (m, 2H, -CH(CH_3_)_2_), 1.34 (d, *J* = 7.4 Hz, 6H, -CH(CH_3_)_2_), 1.31 (d, *J* = 6.8 Hz, 6H, -CH(CH_3_)_2_), 1.18 (d, *J* = 6.7 Hz, 12H, -CH(CH_3_)_2_). ^13^C-NMR (DMSO-*d*_6_) δ (ppm): 181.58 (s, C-4), 158.91 (s, C-7), 158.18 (s, C-4′), 154.34 (s, C-5), 153.90 (s, C-8a), 151.18 (s, C-2), 135.39 (s,C-2′,6′), 125.39 (s, C-1′), 124.41 (s, C-3′,5′), 122.83 (s, C-3), 117.74 (s, C-4a), 112.68 (s, C-6), 105.59 (s, C-8), 30.88 (s, 2C), 26.65 (s, 1C), 24.45 (s, 1C), 23.46 (s, 4C), 21.45 (s, 2C), 20.69 (s, 2C). HRMS (ESI) *m*/*z*: 437.23114 [M − H]^−^, calcd. for C_27_H_3__3_O_5_ 437.23280.

*2′,5′,6,8-Tetraisopropyl genistein* (**2c**). The product was obtained as a white solid (20.3% yield); m.p. 273~274 °C. ^1^H-NMR (DMSO-*d*_6_) δ (ppm): 13.48 (s, 1H, 5-OH), 11.01 (s, 1H, 7-OH), 9.51 (s, 1H, 4′-OH), 8.26 (s, 1H, H-2), 7.07 (s, 1H, H-6′), 6.70 (s, 1H, H-3′), 3.72 (m, *J* = 5.2 Hz, 1H, -CH(CH_3_)_2_), 3.62–3.54 (m, 1H, -CH(CH_3_)_2_), 3.49 (d, *J* = 13.7 Hz, 2H, -CH(CH_3_)_2_), 1.23(d, *J* = 17.4 Hz, 24H, -CH(CH_3_)_2_). ^13^C-NMR (DMSO-*d*_6_) δ (ppm): 172.78 (s, C-4), 159.13 (s, C-7), 158.07 (s, C-4′), 155.39 (s, C-5), 155.11 (s, C-8a), 154.06 (s, C-2), 130.11 (s, C-2′, 6′), 129.78 (s, C-1′), 128.91 (s, C-3), 128.24 (s, C-3′,5′), 105.30 (s, C-4a), 100.36 (s, C-6), 97.40 (s, C-8), 29.62 (s, 2C), 27.08 (s, 2C), 25.68 (s, 1C), 24.46 (s, 1C), 22.68 (s, 1C), 22.46 (s, 1C), 21.48 (s, 2C), 20.66 (s, 2C). HRMS (ESI) *m*/*z*: 437.23457 [M − H]^−^, calcd. for C_27_H_3__3_O_5_ 437.23280.

*2′,6′,6,8-Tetraisopropyl genistein* (**2d**). The product was obtained as a white solid (19.5% yield); m.p. 289~290 °C. ^1^H-NMR (DMSO-*d*_6_) δ (ppm): 13.52 (s, 1H, 5-OH), 11.07 (s, 1H, 7-OH), 9.32 (s, 1H, 4′-OH), 8.25 (s, 1H, H-2), 6.86 (s, 2H, H-3′,5′), 3.63–3.52 (m, 1H, -CH(CH_3_)_2_), 3.53–3.43 (m, 1H, -CH(CH_3_)_2_), 3.16 (dt, *J* = 13.3, 6.8 Hz, 1H, -CH(CH_3_)_2_), 2.66 (dt, *J* = 13.3, 6.6 Hz, 1H, -CH(CH_3_)_2_), 1.37–1.32 (m, 12H), 1.30 (d, *J* = 6.7 Hz, 6H), 1.15 (d, *J* = 6.2 Hz, 6H, -CH(CH_3_)_2_). ^13^C-NMR (DMSO-*d*_6_) δ (ppm): 182.16 (s, C-4), 159.08 (s, C-7), 158.08 (s, C-4′), 155.45 (s, C-5), 155.08 (s, C-8a), 154.07 (s, C-2), 140.08 (s, C-2′,6′), 128.98 (s, C-1′), 125.32 (C-3′,5′), 121.60 (s, C-3), 105.33 (s, C-4a), 96.86 (s, C-6), 93.18 (s, C-8), 34.86 (s, 1C), 30.88 (s, 2C), 30.45 (s, 1C), 26.66 (s, 1C), 24.46 (s, 1C), 22.96 (s, 2C), 21.48 (s, 2C), 20.66 (s, 2C). HRMS (ESI) *m*/*z*: 437.23080 [M − H]^−^, calcd. for C_27_H_3__3_O_5_ 437.23280.

*3′,5′-Diisopropyl* *biochanin A* (**2e**). The product was obtained as a buff solid (21.2% yield); m.p. 216~217 °C. ^1^H-NMR (DMSO-*d*_6_) δ (ppm): 12.94 (s, 1H, 5-OH), 10.90 (s, 1H, 7-OH), 8.37 (s, 1H, H-2), 7.30 (s, 2H, H-2′,6′), 6.41 (d, *J* = 1.5 Hz, 1H, H-8), 6.25 (d, *J* = 1.5 Hz, 1H, H-6), 3.70 (s, 3H, -OCH_3_), 3.29 (dt, *J* = 13.7, 6.9 Hz, 2H, -CH(CH_3_)_2_), 1.22 (d, *J* = 6.9 Hz, 12H, -CH(CH_3_)_2_). ^13^C-NMR (DMSO-*d*_6_) δ (ppm): 180.49 (s, C-4) 165.19 (s, C-7), 162.46 (s, C-4′), 158.08 (s, C-5), 155.12 (s, C-8a), 154.57 (s, C-2), 141.38 (s, C-2′,6′), 127.38 (s, C-1′), 125.41 (s, C-3′,5′), 122.89 (s, C-3), 104.81 (s, C-4a), 99.60 (s, C-6), 94.24 (s, C-8), 62.44 (s, -OCH_3_), 26.52 (s, 2C), 24.34 (s, 4C). HRMS (ESI) *m*/*z*: 367.15324 [M − H]^−^, calcd. for C_22_H_2__3_O_5_ 367.15455.

*3′,5′,8-Triisopropyl* *biochanin A* (**2f**). The product was obtained as a buff solid (19.2% yield); m.p. 211~212 °C. ^1^H-NMR (DMSO-*d*_6_) δ (ppm): 12.99 (s, 1H, 5-OH), 10.87 (s, 1H, 7-OH), 8.45 (s, 1H, H-2), 7.32 (s, 2H, H-2′,6′), 6.33 (s, 1H, H-8), 3.69 (s, 3H, -OCH_3_), 3.28 (dt, *J* = 13.4, 6.6 Hz, 3H, -CH(CH_3_)_2_), 1.21 (d, *J* = 6.7 Hz, 18H, -CH(CH_3_)_2_). ^13^C-NMR (DMSO-*d*_6_) δ (ppm): 180.94 (s, C-2), 162.54 (s, C-7), 159.94 (s, C-4′), 155.55 (s, C-5), 155.08 (s, C-8a), 154.51 (s, C-2), 141.35 (s, C-2′,6′), 127.48(s, C-1′), 125.40 (s, C-3′,5′), 122.34 (s, C-3), 105.61 (s, C-4a), 99.42 (s, C-6), 93.70 (s, C-8), 62.41 (s, -OCH_3_), 26.51 (s, 2C), 25.60 (s, 1C), 24.33 (s, 6C). HRMS (ESI) *m*/*z*: 409.20875 [M − H]^−^, calcd. for C_25_H_29_O_5_ 409.20150.

*3′,5′,6,8-Tetraisopropyl* *biochanin A* (**2g**). The product was obtained as a buff solid (23.4% yield); m.p. 208~209 °C. ^1^H-NMR (DMSO-*d*_6_) δ (ppm): 13.55 (s, 1H, 5-OH), 9.50 (s, 1H, 7-OH), 8.47 (s, 1H, H-2), 7.31 (s, 2H, H-2′,6′), 3.70 (s, 3H,-OCH_3_), 3.62–3.54 (m, 1H, -CH(CH_3_)_2_), 3.53–3.46 (m, 1H, -CH(CH_3_)_2_), 3.32–3.23 (m, 2H, -CH(CH_3_)_2_), 1.40–1.13 (m, 24H, -CH(CH_3_)_2_). ^13^C-NMR (DMSO-*d*_6_) δ (ppm): 181.33 (s, C-4), 159.04 (s, C-7), 158.21 (s, C-4′), 154.89 (s, C-5), 154.40 (s, C-8a), 153.90 (s, C-2), 141.35 (s, C-2′,6′), 127.60 (s, C-1′), 125.38 (s, C-3′,5′), 122.32 (s, C-3), 117.91 (s, C-4a), 112.81 (C-6), 105.59 (s, C-8), 62.44 (s, -OCH_3_), 30.89 (s, 1C), 26.52 (s, 2C), 24.47 (s, 1C), 24.34 (s, 4C), 21.44 (s, 2C), 20.68 (s, 2C). HRMS (ESI) *m*/*z*: 451.24680 [M − H]^−^, calcd. for C_28_H_3__5_O_5_ 451.24845.

*5′-Isopropyl* *biochanin A* (**2h**). The product was obtained as a buff solid (21.2% yield); m.p. 202~203 °C. ^1^H-NMR (DMSO-*d*_6_) δ (ppm): 12.94 (s, 1H, 5-OH), 10.76 (s, 1H, 7-OH), 8.37 (s, 1H, H-2), 7.30 (s, 1H, H-6′), 7.21(d, *J* = 7.7 Hz, 1H, H-2′), 6.88 (d, *J* = 7.8 Hz, 1H, H-3′), 6.42 (s, 1H, H-8), 6.24 (s, 1H, H-6), 3.70 (s, 3H, -OCH_3_), 3.28 (m, *J* = 13.8, 6.9 Hz, 1H, -CH(CH_3_)_2_), 1.36 (d, *J* = 6.9 Hz, 6H, -CH(CH_3_)_2_). ^13^C-NMR (DMSO-*d*_6_) δ (ppm): 180.53 (s, C-4), 164.96 (s, C-7), 162.48 (s, C-4′), 158.07 (s, C-5), 155.16 (s, C-8a), 154.60 (s, C-2), 141.39 (s, C-2′,6′), 128.51 (s, C-1′), 125.41 (s, C-3′,5′), 122.93 (s, C-3), 104.91 (s, C-4a), 99.54 (s, C-6), 94.21 (s, C-8), 62.44 (s, -OCH_3_), 26.53 (s, 1C), 24.34 (s, 2C). HRMS (ESI) *m*/*z*: 325.10345 [M − H]^−^, calcd. for C_19_H_1__7_O_5_ 325.10760.

*3′,5′-Diisopropyl* *formononetin* (**2i**). The product was obtained as a white solid (23.1% yield); m.p. 250~251 °C. ^1^H-NMR (DMSO-*d*_6_) δ (ppm): 10.83 (s, 1H, 7-OH), 8.35 (s, 1H, H-2), 7.97 (d, *J* = 11.2 Hz, 1H, H-5), 7.30 (s, 2H, H-2′,6′), 6.95 (d, *J* = 8.7 Hz, 1H, H-6), 6.88 (s, 1H, H-8), 3.69 (s, 3H, -OCH_3_), 3.28 (dt, *J* = 13.5, 6.7 Hz, 2H, -CH(CH_3_)_2_), 1.26–1.12 (m, 12H, two -CH(CH_3_)_2_).^13^C-NMR (DMSO-*d*_6_) δ (ppm): 175.04 (s, C-4), 163.06 (s, C-7), 157.91 (s, C-4′), 154.36 (s, C-8a), 154.04 (s, C-2), 141.19 (s, C-2′,6′), 128.60 (s, C-5), 127.80 (s, C-1′), 125.31 (s, C-3′,5′), 124.14 (s, C-3), 117.13 (s, C-6), 115.68 (s, C-8), 102.59 (s, C-4a), 62.42 (s, -OCH_3_), 26.50 (s, 2C), 24.36 (s, 4C). HRMS (ESI) *m*/*z*: 351.15826 [M − H]^−^, calcd. for C_22_H_2__3_O_4_ 351.15964.

*3′,5′,8-Triisopropyl formononetin* (**2j**). The product was obtained as a white solid (24.3% yield); m.p. 242~243 °C. ^1^H-NMR (DMSO-*d*_6_) δ (ppm): 10.62 (s, 1H, 7-OH), 8.43 (s, 1H, H-2), 7.85 (d, *J* = 8.6 Hz, 1H, H-5), 7.33 (s, 2H, H-2′,6′), 6.99 (d, *J* = 8.7 Hz, 1H, H-6), 3.74–3.70 (m, 1H, -CH(CH_3_)_2_), 3.70 (s, 3H, -OCH_3_), 3.28 (dt, *J* = 13.4, 6.6 Hz, 2H, -CH(CH_3_)_2_), 1.37 (d, *J* = 6.8 Hz, 6H, -CH(CH_3_)_2_), 1.22 (d, *J* = 6.6 Hz, 12H, -CH(CH_3_)_2_). ^13^C-NMR (DMSO-*d*_6_) δ (ppm): 175.40 (s, C-4), 160.33 (s, C-7), 155.96 (s, C-4′), 154.29 (s, C-8a), 153.92 (s, C-2), 141.16 (s, C-2′,6′), 128.71 (s, C-5), 125.28 (s, C-3′,5′), 124.62 (s, C-1′), 123.47 (s, C-3), 120.69 (s, C-6), 117.51 (s, C-8), 115.21 (s, C-4a), 62.41 (s, -OCH_3_), 26.49 (s, 2C), 24.44 (s, 4C), 24.28 (s, 1C), 20.92 (s, 2C). HRMS (ESI) *m*/*z*: 393.20514 [M − H]^−^, calcd. for C_25_H_29_O_4_ 393.20659.

#### 3.2.3. Synthesis of Compounds **3a**–**3d** (Considering One of the Parental Compounds as an Example)

Treatment of acetic anhydride [19]: P_2_O_5_ was added to acetic anhydride until the P_2_O_5_ became a powder. The mixture was filtered to remove the P_2_O_5_. The filtrate was distilled at 90 °C and 0.096 MPa; fractions were collected at a constant temperature and saved in a dryer.

Treatment of pyridine: KOH was added to pyridine and refluxed for 5 h until the KOH became pasty. Then, the reflux was stopped to distill the solution at a lower pressure. Fractions were collected at 68 °C until the pressure reached 0.085 MPa, and the pyridine was finally sealed with wax.

Synthesis of acetyl ferulic acid: Ferulic acid (19.4 g, 0.1 mol) was dissolved in acetic anhydride (40 mL) and anhydrous pyridine (10 drops). The mixture was stirred at 120 °C for 4 h, and was then cooled to room temperature. A large quantity of precipitate formed immediately. The precipitate was filtered and washed with water and anhydrous ethanol. The resulting solid was finally re-crystallized with anhydrous ethanol, producing the product as a white solid (67.2%); m.p. 190–191 °C.

Synthesis of target compounds **3a**–**3d**: Acetyl ferulic acid (0.4 g, 1.69 mmol), genistein (0.5 g, 1.85 mmol), and DCC (0.6 g, 2.90 mmol) were dissolved in tetrahydrofuran (20 mL), and anhydrous pyridine (1 mL) was then added to the mixture. The mixture was then stirred at 80 °C for 4 h. The resulting mixture was filtered to remove the DCU, and the crude material was then purified in a 300–400 mesh silica column (petroleum ether:ethyl acetate:methanol, 6:2:1).

*Genistein 7-acetylferulic acid* (**3a**). The product was obtained as a white solid (33.2%); m.p. 257~258 °C. ^1^H-NMR (DMSO-*d*_6_) δ (ppm): 13.02 (s, 1H, 5-OH), 9.63 (s, 1H, 4′-OH), 8.52 (s, 1H, H-2), 7.92 (d, 1H, *J* = 15.4 Hz, H-3′′), 7.63 (m, 1H, H-2′′′), 7.45 (d, 2H, *J* = 8.3 Hz, H-2′,6′), 7.17 (m, 1H, H-6′′′), 7.13 (m, 1H, H-5′′′), 6.97 (d, 1H, *J* = 15.4Hz, H-2′′), 6.83 (d, 2H, *J* = 8.3 Hz, H-3′,5′), 6.81 (s, 1H, H-6), 6.77 (s, 1H, H-8), 3.86 (s, 3H, -OCH_3_), 2.33 (s, 3H, -COCH_3_); ^13^C-NMR (DMSO-*d*_6_) δ (ppm): 180.7 (s, C-4), 168.2 (s, C-1′′), 164.2 (s, -COO-), 161.1 (s, C-5), 157.8 (s, C-8a), 156.6 (s, C-7), 155.7 (s, C-4′), 155.6 (s, C-2), 151.8 (s, C-3′′′), 146.1 (s, C-3′′), 140.3 (s, C-4′′′), 132.9 (s, C-1′′′), 131.2 (s, C-2′,6′), 123.4 (s, C-1′), 123.1 (s, C-5′′′), 122.0 (s, C-3), 120.4 (s, C-6′′′), 116.3 (s, C-2′′′), 115.3 (s, C-3′, 5′), 112.2 (s, C-2′′′), 108.4 (s, C-4a), 104.2 (s, C-6), 101.1 (s, C-8), 56.4 (s, -OCH_3_), 20.5 (s, -CH_3_). HRMS (ESI) *m*/*z*: 489.10428 [M + H]^+^, calcd. for C_27_H_21_O_9_ 489.11856.

*Genistein 7,4′-diacetylferulic acid* (**3b**). The product was obtained as a white solid (31.7%); m.p. 234–235 °C. ^1^H-NMR (DMSO-*d*_6_) δ (ppm): 12.95 (s, 1H, 5-OH), 8.73 (s, 1H, H-2), 7.97 (d, 2H, *J* = 15.64 Hz, H-3′′), 7.75 (m, 2H, H-2′′′), 7.71 (d, 2H, *J* = 8.35 Hz, H-2′,6′), 7.43 (d, 2H, *J* = 8.35 Hz, H-3′,5′), 7.42 (m, 2H, H-6′′′), 7.22 (m, 2H, H-5′′′), 7.05 (d, 2H, *J* = 15.65 Hz, H-2′′), 6.85 (s, 1H, H-6), 6.84 (s, 1H, H-8), 3.91 (s, 6H, -OCH_3_), 2.33 (s, 6H, -CH_3_); ^13^C-NMR (DMSO-*d*_6_) δ (ppm): 180.1 (s, C-4), 167.5 (s), 167.7 (s, C-1′′), 164.2 (s), 164.2 (s, 2-COO-), 156.2 (s, C-5), 155.7 (s, C-4′), 152.4 (s, C-7), 150.5 (s, C-8a), 150.5 (s), 150.6 (s, C-3′′′), 150.2 (s, C-2), 145.3 (s), 145.6 (s, C-3′′), 141.3 (s), 141.3 (s, C-4′′′), 131.6 (s), 132.1 (s, C-1′′′), 129.2 (s, C-2′,6′), 122.6 (s, C-1′), 122.3 (s), 122.3 (s, C-5′′′), 120.8 (s, C-3), 120.5 (s), 120.6 (s, C-6′′′), 116.4 (s), 116.6 (s, C-2′′), 112.4 (s, C-3′,5′), 110.6 (s), 110.7 (s, C-2′′′), 104.7 (s, C-4a), 100.1 (s, C-6), 95.3 (s, C-8), 55.1, 55.1 (s, -OCH_3_), 19.4 (s), 19.4 (-CH_3_). HRMS (ESI) *m*/*z*: 707.15763 [M + H]^+^, calcd. for C_39_H_31_O_13_ 707.17647.

*Biochanin A 7-acetylferulic acid ester* (**3c**). The product was obtained as a buff solid (38.3%); m.p. 192~193 °C. ^1^H-NMR (DMSO-*d_6_*) δ (ppm): 12.86 (s, 1H, 5-OH), 8.46 (s, 1H, H-2), 7.61 (d, *J* = 8.4 Hz, 2H, H-2′,6′), 7.38 (d, *J* = 8.4 Hz, 1H, H-6′′′), 7.21 (d, *J* = 8.4 Hz, 2H, H-3′,5′), 7.05–6.97 (d, *J* = 16.4 Hz, 1H, H-3′′), 6.88 (s, 1H, H-2′′′), 6.82 (d, *J* = 8.4 Hz, 1H, H-5′′′), 6.75–6.66 (d, *J* = 16.1 Hz, 1H, H-2′′), 6.41 (s, 1H, H-8), 6.25 (s, 1H, H-6), 3.80 (s, 3H, -OCH_3_), 3.93 (s, 3H, -OCH_3_), 2.30 (s, 3H, CH_3_COO-). ^13^C-NMR (DMSO-*d*_6_) δ (ppm): 180.28 (s, C-4), 169.69 (s, C-1′′), 165.14 (s, -COO-), 162.45 (s, C-5), 158.09 (s, C-8a), 156.69 (s, C-7), 155.50 (s, C-4′), 154.45 (s, C-2), 151.94 (s, C-3′′′), 150.78 (s, C-3′′), 139.66 (s, C-4′′′), 133.3 (s, C-1′′′), 130.59 (s, C-2′,6′), 128.85 (s, C-1′), 125.38 (s, C-5′′′), 122.74 (s, C-3), 122.17 (s, C-6′′′), 115.60 (s, C-2′′), 115.53 (s, C-3′,5′), 111.74 (s, C-2′′′), 106.01(s, C-4a), 99.67 (s, C-6), 94.34 (s, C-8), 57.44 (s, -OCH_3_), 49.94 (s, -OCH_3_), 21.93 (s, -CH_3_). HRMS (ESI) *m*/*z*: 503.11478 [M + H]^+^, calcd. for C_28_H_23_O_9_ 503.13421.

*Formononetin 7-acetylferulic acid ester* (**3d**). The product was obtained as a white solid (30.8%); m.p. 236~237 °C. ^1^H-NMR (DMSO-*d*_6_) δ (ppm): 8.44 (s, 1H, H-2), 8.04 (d, *J* = 8.8 Hz, 1H, H-5), 7.66 (d, *J* = 16.3 Hz, 1H, H-3′′), 7.62 (s, 1H, H-2′′′), 7.54 (d, *J* = 6.2 Hz, 2H, H-2′,6′),7.37 (d, *J* = 8.0 Hz, 1H, H-6′′′), 7.32 (d, *J* = 8.4 Hz, 1H, H-5′′′), 7.08 (d, *J* = 8.9 Hz, 1H, H-6), 7.01 (d, *J* = 7.3 Hz, 2H, H-3′,5′), 6.88 (s, 1H, H-8), 6.73 (d, *J* = 16.3 Hz, 1H, H-2′′), 3.87 (s, 3H, -OCH_3_), 3.79 (s, 3H, -OCH_3_), 2.30 (d, *J* = 8.5 Hz, 3H, -COCH_3_).^13^C-NMR (DMSO-*d*_6_) δ (ppm): 180.91 (s, C-4), 168.63 (s, C-1′′), 164.91 (s, -COO-), 162.10 (s, C-7), 157.93 (s, C-4′), 156.27 (s, C-8a), 152.03 (s, C-2), 150.36 (s, C-3′′′), 146.21 (s, C-1′′), 142.04 (s, C-4′′′), 132.50 (s, C-1′′′), 130.66 (s, C-2′,6′), 128.62 (s, C-5), 125.39 (s, C-1′), 124.80 (s, C-3), 123.70 (s, C-5′′′), 119.96 (s, C-6′′′), 117.95 (s, C-2′′′), 116.60 (s, C-6), 114.92 (s, C-8), 114.09 (s, C-3′,5′), 112.34 (s, C-2′′′), 101.43 (C-4a), 64.71 (s, -OCH_3_), 56.61 (s, -OCH_3_), 21.89 (s, -CH_3_). HRMS (ESI) *m*/*z*: 509.12245 [M + Na]^+^, calcd. for C_28_H_2__2_NaO_8_ 509.12124.

#### 3.2.4. Synthesis of Compounds **4a**–**4e** (Take One of the Parental Compounds as an Example)

Formononetin (0.6 g, 2.1 mmol) was dissolved in anhydrous methanol (60 mL), and CH_3_ONa-CH_3_OH (5 mL) was then added to the mixture. 4-fluorobenzyl bromide (0.47 g, 2.5 mmol) was dissolved in dichloromethane (10 mL) and then added to the mixture. The solution was stirred under reflux for 20 h. The resulting product was filtered and separated in a 300–400 mesh silica column (petroleum ether:ethyl acetate:methanol, 6:2:1).

*7-(4-Fluorine benzyl)-O-genistein* (**4a**). The product was obtained as a white solid (30.1%); m.p. 243~244 °C. ^1^H-NMR (DMSO-*d*_6_) δ (ppm): 12.95 (s, 1H, 5-OH), 9.63 (s, 1H, 4′-OH), 8.45 (s, 1H, H-2), 7.57–7.49 (m, 2H, H-2′′,6′′), 7.40 (d, *J* = 8.0 Hz, 2H, H-2′,6′), 7.25 (d, *J* = 8.5 Hz, 2H, H-3′′,5′′), 6.83 (d, *J* = 7.9 Hz, 2H, H-3′,5′), 6.76 (s, 1H, H-8), 6.50 (s, 1H, H-6), 5.19 (s, 2H, -CH_2_-). ^13^C-NMR (DMSO-*d*_6_) δ (ppm): 180.89 (s, C-4), 164.56 (s, C-7), 163.39 (s, C-4′′), 162.24 (s, C-4′), 157.93 (s, C-5), 157.90 (s, C-8a) 154.96 (s, C-2), 132.80 (s, C-1′′), 130.83 (s, C-2′′), 130.76 (s, C-6′′), 130.66 (s, C-2′,6′), 122.99 (s, C-1′), 121.49 (s, C-3), 115.96 (s, C-3′′), 115.78 (s, C-5′′), 115.55 (s, C-3′,5′), 106.02 (s, C-4a), 99.14 (s, C-6), 93.77 (s, C-8), 69.77 (s, -CH_2_O-). HRMS (ESI) *m*/*z*: 377.09124 [M − H]^−^, calcd. for C_22_H_1__4_FO_5_ 377.08253.

*7-(4-Fluorine benzyl)-O-biochanin* (**4b**). A The product was obtained as a buff solid (26.8%); m.p. 175~176 °C. ^1^H-NMR (DMSO-*d*_6_) δ (ppm): 12.94 (s, 1H, 5-OH), 8.48 (s, 1H, H-2), 7.54 (dd, *J* = 13.6, 7.4 Hz, 4H, H-2′,6′; H-2′′,6′′), 7.26 (t, *J* = 8.3 Hz, 2H, H-3′′,5′′), 7.02 (d, *J* = 7.6 Hz, 2H, H-3′,5′), 6.78 (s, 1H, H-8), 6.52 (s, 1H, H-6), 5.23 (s, 2H, -CH_2_-), 3.80 (s, 3H, -OCH_3_). ^13^C-NMR (DMSO-*d*_6_) δ (ppm): 180.81 (s, C-4), 167.45 (s, C-4′′), 164.62 (s, C-7), 162.24 (s, C-4′), 159.69 (s, C-5), 157.92 (s, C-8a), 155.30 (s, C-2), 132.02 (s, C-1′′), 130.84 (s, C-2′′), 130.78 (s, C-6′′), 130.66 (s, C-2′,6′), 123.21 (s, C-1′), 122.67 (s, C-3), 115.96 (s, C-3′′), 115.79 (s, C-5′′), 114.21 (s, C-3′,5′), 106.02 (s, C-10), 99.21 (s, C-6), 93.84 (s, C-8), 69.79 (s, -CH_2_O-), 55.65 (s, CH_3_O-). HRMS (ESI) *m*/*z*: 391.11194 [M − H]^–^, calcd. for C_23_H_1__6_FO_5_ 391.09818.

*7-(4-Fluorine benzyl)-O-formononetin* (**4c**). The product was obtained as a white solid (32.3%); m.p. 193~194 °C. ^1^H-NMR (DMSO-*d*_6_) δ (ppm): 8.44 (s, 1H, H-2), 8.05 (d, *J* = 8.8 Hz, 1H, H-5), 7.62–7.48 (m, 4H, H-2′,6′; H-2′′,6′′), 7.23–7.33 (m, 3H, H-3′′,5′′;H-8), 7.16 (d, *J* = 8.8 Hz, 1H, H-6), 7.01 (d, *J* = 7.6 Hz, 2H, H-3′,5′), 5.26 (s, 2H, -CH2-), 3.80 (s, 3H, -OCH_3_). ^13^C-NMR (DMSO-*d*_6_) δ (ppm): 175.09 (s, C-4), 167.44 (s, C-4′′), 163.06 (s, C-7), 159.48(s, C-4′), 157.81 (s, C-8a), 154.03 (s, C-2), 132.02 (s, C-1′′), 130.89 (s, C-2′′), 130.82 (s, C-6′′), 130.56 (s, C-2′, 6′), 127.50 (s, C-5), 124.50 (s, C-1′), 123.85 (s,C-3), 118.23 (s, C-6), 115.97 (s, C-8),115.80 (s, C-3′′), 115.73(s, C-5′′), 114.10 (s, C-3′,5′), 102.06 (s, C-4a), 69.82 (s, -CH_2_O-), 55.62 (s, CH_3_O-). HRMS (ESI) *m*/*z*: 399.11270 [M + Na]^+^, calcd. for C_2__3_H_17_FNaO_4_ 399.10085.

#### 3.2.5. Synthesis of Compound **5**

Biochanin A (0.81 g, 2.8 mmol) was dissolved in anhydrous ethanol (30 mL). The mixture was heated to 60 °C. After the biochanin A dissolved, (CH_3_COO)_3_Cr (0.34 g, 1.5 mmol) was added to the solution, and the mixture was stirred under reflux for 18 h. The mixture was then cooled to room temperature. A green precipitate formed and was filtered. The precipitate was washed with DI H_2_O and anhydrous ethanol until the biochanin A and (CH_3_COO)_3_Cr were removed. The resulting product was purified in a 300–400 mesh silica column (petroleum ether:ethyl acetate:methanol, 6:2:1).

(1) The physicochemical properties and element analysis

The biochanin A chromium complex was in the form of a grass-green powder that could be dissolved in DMSO, dimethyl formamide (DMF), and tetrahydrofuran (THF), that was slightly soluble in methanol or acetone and that was insoluble in water and CCl_4_. The C and H contents of the biochanin A chromium complex were measured using a Vario EL element analyzer. The chromium content was measured via EDTA titration. The molecular weight was analyzed using a Thermo LQT Orbitrap XL LC-MS. The results are shown in Table 2.

(2) Thermogravimetry (TG) and differential thermal analysis (DTA)

The thermal spectrum of the complex was measured under airflow at a heating rate of 10 °C/min. Two stages of weight loss can be observed in the TG-DTA spectrum. The first stage of weight loss was at approximately 75 °C, corresponding to a weight loss rate of 3.94% and a considerable loss of 2 water molecules (the theoretical weight loss rate was 4.05%). The corresponding absorption peak in the DTA curve was very small and showed that the complex contained 2 crystal water molecules. The second stage included the range from 320 °C to 420 °C, and the weight loss rate was 90.60%, corresponding to the considerable loss of 3 ligand molecules (the theoretical weight loss rate was 90.62%). The corresponding absorption peak, which was an oxidation-decomposition peak of the complex in the DTA curve, was very large. Chromium was oxidized in air, and the remnant of the decomposition was Cr_2_O_3_.

**Table 2:** Elemental analysis of the complex.

C %		H %		Cr %		Molecular	
test	theory	test	theory	test	theory	test	theory [M − 2H_2_O − H]^−^
61.43	61.47	3.93	3.95	5.53	5.55	900.12354	900.11463

(3) Infrared spectrum analysis

**Figure 7:**
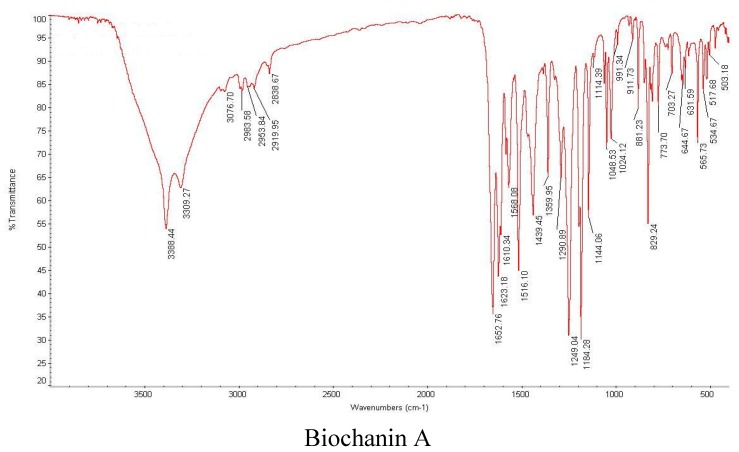

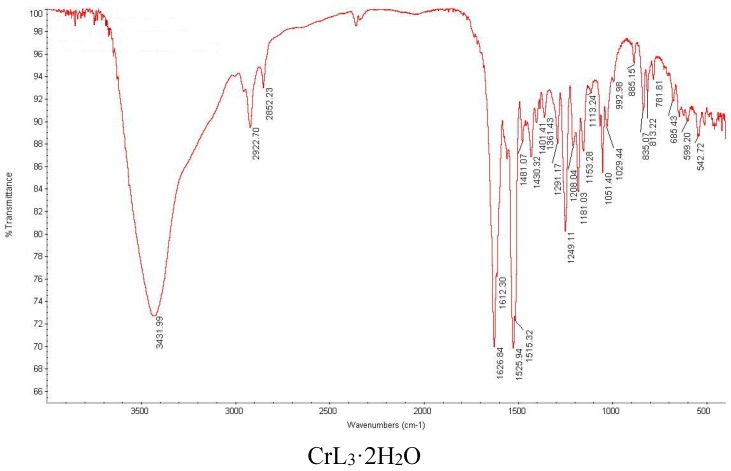
IR spectra analysis.

From 400–4000 cm^−1^, the IR spectra of the ligand and complex were measured using a Thermo-Nicolet nexus of a Fourier transform IR spectrometer (KBr pellets), as shown in Figure 7. The primary spectral data are listed in Table 3.

A comparison of the IR spectral data of the ligand and chromium complex is shown in Table 3. Biochanin A exhibited an absorption band (OH) at 3388 cm^−1^, which shifted to 3432 cm^−1^ in the IR spectrum of the complex. Biochanin A exhibited a very strong absorption band at 1653 cm^−1^ due to the stretching vibration of the 4-carbonyl, which shifted to 1627 cm^−1^ in the IR spectrum of its complex. Therefore, coordination of the 4-carbonyl is involved in the coordination of the ligands.

**Table 3:** The primary IR spectral data of genistein and the chromium complexes (cm^−1^).

Compound	V(O-H)	V(C=O)	V(C=C)	V(C-O-C)
Biochanin A (L)	3388	1653	1623	1249
CrL_3_·2H_2_O	3432	1627	1612	1249

(4) Ultraviolet spectrum

**Figure 8:**
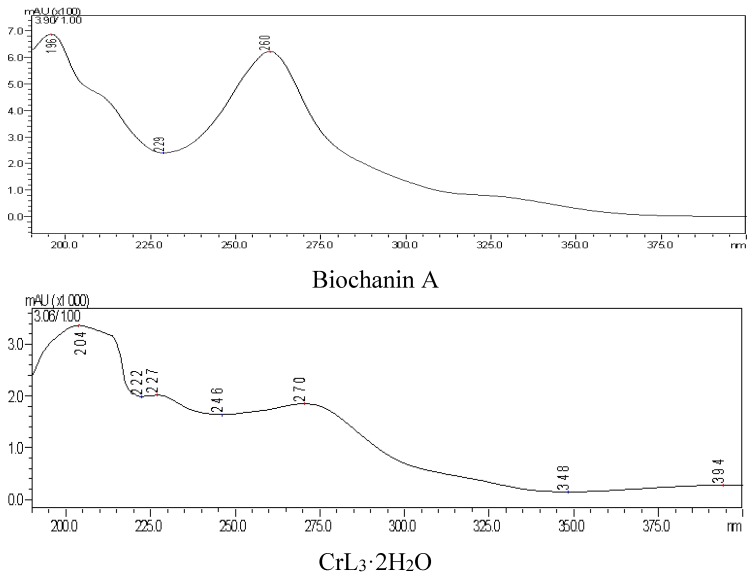
UV spectra analysis.

There were two characteristic UV absorption peaks at 260 nm (band II) and 330 nm (band I, weak) in DMSO:CH3OH = 1:9 solvent. After complex formation, the two peaks red shifted to 270 nm and 394 nm (weak). Because the B ring could not conjugate with the unsaturated carbonyl of the C ring in biochanin A, the absorption intensity of the band I was therefore weakened. However, the band I was red-shifted in the chromium complex, and the intensity of this band increased due to the enhanced plane type of molecule. The conjugated system of complex was larger, which was attributed to the 4-carbonyl and 5-hydroxyl of the three biochanin A molecules coordinated with chromium, as shown in Figure 8.

### 3.3. Biological Activity

#### 3.3.1. Hypoglycemic Activity Screening

Cell culture and IR-HepG2 model [20,21]: HepG2 cells were cultured in high-glucose Dulbecco’s modified Eagle’s medium (DMEM) supplemented with 10% fetal bovine serum (FBS). After reaching confluence, the cells were cultured in 96-well cluster plates with density of 10^4^ cells/mL in high-glucose DMEM supplemented with 10% FBS for 24 h and then treated with 10^−7^ mol/L insulin for 36 h in serum-free medium. After 36 h of stimulation with a high concentration of insulin, the cells were washed twice with phosphate-buffered saline (PBS). Then serum-free high-glucose DMEM was added with compounds in different concentrations and incubated for 24 h. After 24 h, the glucose content in the culture medium was measured using a glucose assay kit to study the effect of IR-HepG2 cells on glucose consumption. The enhancement ratio of glucose consumption (GC) was calculated as follows: GC% = (GC of the drug group-GC of the model group)/GC of the model group × 100. The ED_50_ values were calculated using GraphPad Prism 5.

**Figure 9:**
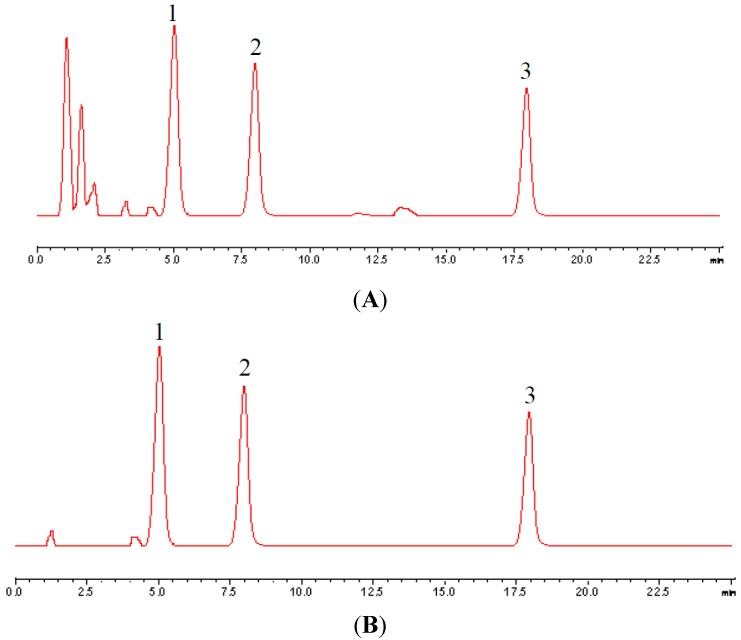
Chromatograms of test sample (**A**) and reference substances (**B**).

We then combined the derivatives (**1c**, **2h**, **3b**, and **5**) and genistein (instead of G) to attempt to obtain improved hypoglycemic activities using these combinations. The derivatives were dissolved in a certain volume of DMEM. The concentration of each compound in these combinations was 1/3 the quantity of the original concentration. This concentration was determined which primarily based on our previous study of the three compounds in chickpea. Wherein the content of genistein and biochanin A was basically 1: 1 relationship, as shown in Figure 9 and Table 4. Test sample (A) and reference substances (B), 1 genistein; 2 biochanin A; 3 formononetin.

**Table 4:** Result of content determinition of five samples (mg∙g^−1^, *n* = 5, *x* ± *s*).

Lot No.	Genistein	Biochanin A	Formononetin
141205	1.42 ± 0.13	1.35 ± 0.08	1.15 ± 0.08
141208	1.41 ± 0.11	1.38 ± 0.07	1.12 ± 0.09
141212	1.45 ± 0.12	1.31 ± 0.09	1.21 ± 0.08
141218	1.48 ± 0.15	1.39 ± 1.11	1.13 ± 0.07
141220	1.42 ± 0.09	1.34 ± 1.15	1.18 ± 0.09

Cell morphology was observed using an optical microscope. All the images were captured at the maximum concentration of the compounds and before determination. The cellular morphology was assessed by green light microscopy (Nikon, Kobe, Japan).

#### 3.3.2. Combination 6 and Combination 10 Acting on HUVEC under High Concentration of Glucose

HUVEC were cultured in endothelial cell medium (ECM). ECM contains 5% fetal bovine serum, penicillin/streptomycin solution, endothelial cell growth supplements (ECGS), and d-glucose concentration is 5.55 mmol/L. The HUVEC were inoculated in the ECM after recovery and cultured at 37 °C, 5% CO_2_ in the incubator. When the cells growed to 80% confluence, the cells were digested with 0.025% trypsin-EDTA 1min after washing with PBS 2 times. And then added trypsin neutralize solution when 80% of the cells became round. Pipetting cells into single, and inoculated into 25 cm^2^ cell culture flasks after centrifugation. The cells were uesed into experimental study after 4–8 generations passed. The cells were cultured in 96-well cluster plates with density of 10^5^ cells/mL and cultured in 37 °C, 5% CO_2_ incubator for 24 h. Then serum-free culture medium DMEM 199 with low concentration of sugar was used to culture for 24 h so that cells were in a stationary state. Then ECM (The concentration of glucose was 25 mmol/L) was added with compounds in different concentrations and incubated for 48 h (This time point was determined through the pre-test results for the maximum difference between control group and test group). The LDH, MDA, SOD and NO were measured after cell supernatant liquid was collected.

##### Detection method

(1) Determination of MDA content

The MDA content was tested according to kit instructions to MDA by TBA method. 20 μL cell supernatant liquid from each well was used to determine.

(2) Detection of LDH release quantity

LDH release quantity was tested according to kit instructions of LDH. 20 μL cell supernatant liquid from each well was used to determine.

(3) Detection of NO content

The NO content was tested according to kit instructions of NO by nitrate reductase method. 20 μL cell supernatant liquid from each well was used to determine.

(4) Determination of SOD activity

The SOD activity was tested according to kit instructions by Xanthine oxidase method. 10 μL cell supernatant liquid from each well was used to determine.

## 4. Conclusions

In this study, fifteen novel isoflavonoid derivatives from chickpea were synthesized and characterized by ^1^H-NMR, ^13^C-NMR and MS analyses. The preliminary bioassay data indicated that the target compounds **1c**, **2h**, **3b**, and **5** exerted increased anti-hyperglycemic activities compared with parent compounds. In addition, the combination of genistein, **2h**, **3b** and of **3b**, genistein, **1c** exhibited better anti-diabetic activity than other combinations. At the same dosage as the positive control, combination 10 exerted the same anti-diabetic activity as the positive control (*p* > 0.05). These results showed that the combinations could act on many targets in the body simultaneously and that stronger effects could be induced by combining compounds.

The endothelial cell is a multifunctional cell. It regulates vascular tone, and maintains the balance between coagulation and fibrinolysis system, and regulates the accumulation of inflammatory cells, and suppresses the aggregation and activation of platelet [22]. The dysfunction of endothelial cells is an important factor leading to vascular and nerve disease of diabetes [23]. Research reported high sugar concentration could cause damage to endothelial cells, causing MDA, LDH, NO, SOD and other abnormalities [24]. Combination 10 had protective effect on high glucose injured HUVEC. It may be related to the regulation of cell activity, improve oxidative stress, maintain cell membrane stability. This result provided a reference for better prevention and treatment of diabetes.

Further biological evaluations and the mechanism of the active compounds are currently underway.
